# Greater Biofilm Formation and Increased Biodegradation of Polyethylene Film by a Microbial Consortium of *Arthrobacter* sp. and *Streptomyces* sp.

**DOI:** 10.3390/microorganisms8121979

**Published:** 2020-12-12

**Authors:** Ya-Nan Han, Min Wei, Fang Han, Chao Fang, Dong Wang, Yu-Jie Zhong, Chao-Li Guo, Xiao-Yan Shi, Zhong-Kui Xie, Feng-Min Li

**Affiliations:** 1 State Key Laboratory of Grassland Agro-Ecosystems, Institute of Arid Agroecology, School of Life Sciences, Lanzhou University, Lanzhou 730000, China; hanyn15@lzu.edu.cn (Y.-N.H.); weim@lzu.edu.cn (M.W.); hanf19@lzu.edu.cn (F.H.); wangd16@lzu.edu.cn (D.W.); zhongyj19@lzu.edu.cn (Y.-J.Z.); guochl19@lzu.edu.cn (C.-L.G.); shixiaoyan@lzu.edu.cn (X.-Y.S.); 2 Northwest Institute of Eco-Environment and Resources, Chinese Academy of Sciences, No. 320 West Donggang Road, Lanzhou 730000, China; wxhcas@lzb.ac.cn; 3 University of Chinese Academy of Sciences, Beijing 100049, China; 4 Institute of Ecology, School of Applied Meteorology, Nanjing University of Information Science and Technology, Nanjing 210044, China; fangch12@lzu.edu.cn

**Keywords:** *Arthrobacter*, carbonyl index, FTIR spectroscopy, microbial consortium, plastic biodegradation, plastic pollution, *Streptomyces*, SEM, water contact angle

## Abstract

The widespread use of polyethylene (PE) mulch films has led to a significant accumulation of plastic waste in agricultural soils. The biodegradation of plastic waste by microorganisms promises to provide a cost-effective and environmentally-friendly alternative for mitigating soil plastic pollution. A large number of microorganisms capable of degrading PE have been reported, but degradation may be further enhanced by the cooperative activity of multiple microbial species. Here, two novel strains of *Arthrobacter* sp. and *Streptomyces* sp. were isolated from agricultural soils and shown to grow with PE film as a sole carbon source. *Arthrobacter* sp. mainly grew in the suspension phase of the culture, and *Streptomyces* sp. formed substantial biofilms on the surface of the PE film, indicating that these strains were of different metabolic types and occupied different microenvironments with contrasting nutritional access. Individual strains were able to degrade the PE film to some extent in a 90-day inoculation experiment, as indicated by decreased hydrophobicity, increased carbonyl index and CO_2_ evolution, and the formation of biofilms on the film surface. However, a consortium of both strains had a much greater effect on these degradation properties. Together, these results provide new insights into the mechanisms of PE biodegradation by a microbial consortium composed of different types of microbes with possible metabolic complementarities.

## Highlights

- Two novel soil bacterial strains of *Arthrobacter* sp. and *Streptomyces* sp. were isolated and shown to be capable of growing on polyethylene (PE) film as a sole carbon source.
- Both strains could form biofilms on the PE film surface, thereby increasing film hydrophilicity, carbonyl index, and mineralization.
- Inoculation with their consortium resulted in a much thicker and more complex biofilm and had significantly greater effects on film hydrophilicity, surface chemistry, and mineralization.

## 1. Introduction

Agricultural mulch film is widely used to cover cultivated fields, where it promotes higher soil temperatures [1], better soil moisture conservation [2], effective weed suppression [3], and increased crop yields [4]. Its excellent physical and mechanical properties and affordable price make plastic mulch film the most indispensable and highly consumed product [5,6], with an estimated 1.25−1.4 million tons of film applied annually in China [7,8]. Most plastic mulch film in China is made primarily of polyethylene (PE) with superior properties such as high molecular weight (>30 kDa), 3D structure, and hydrophobicity, but these properties make PE highly resistant to degradation [9]. Recently, the government has established waste film recycling laws, but thin and ultra-thin agricultural mulch films are rarely recycled in practice. Very large amounts of plastic film residues that cannot be recovered are therefore left on the soil after use [10], and the residual amount of plastic film in soil was about 50–250 kg/ha [11]. Hence, plastic film residues remain quasi-permanently in the soil [12], a situation that is not compatible with sustainable agricultural practices. The potential risks associated with these film residues are a cause for serious environmental concern in agroecosystems [13]. Thus, it is essential to develop an efficient method to degrade such plastics.

Numerous research programs are attempting to degrade plastics with microorganisms. Plastic biodegradation by means of enzymes secreted from microorganisms may present a promising, cost-effective means of plastic degradation [14]. A number of PE-degrading microbes have been isolated from various environments, such as soil, seawater, sludge, compost, and the insect gut [15]. For example, Yang et al. [16] isolated two PE-degrading bacterial strains (*Enterobacter asburiae* YT1 and *Bacillus* sp. YP1) from the worm gut. A strain of *Rhodococcus ruber* (C208) was isolated from soil that contained buried PE waste and was shown to degrade 8% of the initial PE dry weight over 30 days of incubation [17]. Studies on the biodegradation of PE microplastics by the marine fungus Zalerion maritimum showed that it could use PE as a substrate, resulting in decreased mass and size of PE pellets [18].

It is generally accepted that microbial biofilm formation on the polymer surface is a prerequisite for biodegradation [19]. After initial attachment of microorganisms to material surfaces, they can form extensive biofilms [20] and may consequently alter the physicochemical properties of plastic film, including changes in functional groups, hydrophobicity/hydrophilicity, crystallinity, surface morphology, and molecular weight distribution. Measurements have indicated that film surfaces become more hydrophilic after microorganism attachment [21]; however, polymer hydrolyzes at a rate much faster than the degradation triggered by microorganisms [22]. Plastic surface topography can be modified after bacterial inoculation, and pits and cavities have been observed on the polymer surface [23]. PE film incubated with *Comamonas* sp. and *Delftia* sp. showed a decrease in crystallinity, whereas film incubated with *Stenotrophomonas* sp. displayed an increased crystalline fraction [24]. Findings on the changes in polymer molecular weight (number-averaged and weight-averaged molecular weight (Mn and Mw, respectively) appear to be contradictory. For example, some studies claim that molecular weight decreased after bacterial inoculation, but others reported that it increased after exposure to bacteria [25]. Transformation of functional groups can be determined by Fourier transform infrared (FTIR) spectroscopy analyses. Increase or decrease of functional groups is influenced by the aerobic or anaerobic milieu. Under aerobic conditions, some studies have documented an increase in functional groups after microbial exposure [26], whereas others have reported a decrease [27]. Hence, conflicting changes in PE film after microbial exposure have been reported, and a better understanding of the interactions between plastics and microbes may improve efforts aimed at the mitigation of plastic pollution.

Numerous studies have demonstrated the ability of pure strains to degrade polymers, and the use of a pure microbial strain is convenient for investigating metabolic pathways and evaluating the effects of different environmental conditions on PE degradation [28]. However, this approach ignores the possibility that PE biodegradation may result from the cooperative activity of different microbial species under natural conditions, and the activity of a single species may lead to the accumulation of intermediates or dead-end products with potentially higher toxicity, which inhibits microbial growth [29]. Complex mixtures of compounds may have regulatory and inhibitive effects on biodegradation, leading to temporary accumulation or a lack of degradation of single substances or substance classes [30]. This limitation can be overcome by using microbial consortia, in which toxic metabolites produced by one microbe can often be used as a substrate for the growth of another [24]. The bacteria in consortia have synergistic interactions, allowing them to have increased tolerance towards the biodegradation of PE [31].

The aim of the present study was to isolate various soil microbes that exhibited a potential ability to degrade plastic film. Two novel soil bacteria were isolated and found to grow in medium with plastic films as the sole carbon source. The presence of each strain effectively altered the degradation properties of the plastic film. Furthermore, a markedly synergistic ability to degrade plastic film was observed for the consortium of both strains, providing insights into the biodegradation of polymers by microbiota.

## 2. Materials and Methods

### 2.1. Materials

Unpretreated plastic film (UPF) (6 μm thickness) was kindly provided by Lanzhou Lvyuan Co., Ltd. (Gansu, China). This material is composed of linear low-density polyethylene (LLDPE, DFDA7042), low-density polyethylene (LDPE, 2426F), and functional masterbatch at a weight ratio of 47.5:1:1. Plastic films (10 μm thickness) exposed to UV radiation under natural weathering conditions for five months (1 May, to 1 October, 2019) during the mulching of corn were defined as weathered plastic film (WPF), and plastic films (6 μm thickness) buried in the soil for 30 months (1 April, 2016 to 1 October, 2018) were defined as buried plastic film (BPF). UPF and WPF were also cut into small pieces (ca. 1 mm × 1 mm) for carbon dioxide (CO_2_) analysis. To obtain powders, the LLDPE, LDPE, and functional masterbatch particles were crushed separately with a pulverizer and then passed through a 0.157 mm sieve. The PE or functional masterbatch powders were used as sole carbon sources in the CO_2_ evolution experiment.

### 2.2. Culture Enrichment, Isolation, and Preliminary Screening of Polyethylene-Degrading Bacteria

To isolate the polymer-degrading microorganisms, Czapek–Dox medium [32] and liquid carbon-free basal medium (LCFBM) [16] containing PE powder or PE film as the sole carbon source were used throughout the study. Soil plastic film residues with strong evidence of degradation such as cracks and holes were collected in Yuzhong, Gansu province, China and were used to enrich the PE-degrading bacteria. In detail, the film residues were sampled, placed in 50-mL sterile tubes, and transported to the laboratory in ice boxes. Then, they were added to 150-mL Erlenmeyer flasks with 100 mL of the broth media described above. The flasks were first shaken at 120 rpm for 10 days. Then, 1 mL of suspended solution was transferred to another 100 mL fresh medium, and 0.3 g PE film powder (pre-sterilized by exposure to UV radiation for 2 h) was added. The cultures were then spread repeatedly onto agar medium containing PE powder to obtain pure strains. For further preliminary screening of PE degraders, isolated microbes were grown in fresh liquid medium containing PE film powder for 90 days, and the surviving microbes were stored at −80 °C in 20% glycerol. The same procedure (preliminary screening) was repeated once again to ensure that the isolated microbe had the ability to use PE film as the sole carbon source to grow in long-term experiments. All bacterial cultures were incubated at 25 °C in a rotary shaker at 120 r/min during the study.

### 2.3. Bacterial Growth Measurement

Microbial growth was measured using the serial dilution method of plate counting. Colony-forming units (CFU) of planktonic microbes were counted after incubation periods of 0, 15, 30, 45, 60, 75, and 90 days. After 90 days, bacteria that adhered to the PE films (i.e., bacterial biofilms) were measured using the following procedure. The PE film was removed from the medium, gently rinsed with sterile phosphate buffer (pH 7) to eliminate loosely adhering bacteria, and washed for 5 min in sterile phosphate buffer by vortexing three times. Suspensions were collected from three tubes, and the cells were harvested by centrifugation at 8000× *g* for 5 min. The cell pellets were suspended and combined in 1 mL sterile phosphate buffer, and the cell numbers were counted by the conventional spreading plate-culture method.

### 2.4. Analytical Techniques

#### 2.4.1. Hydrophobicity

Contact angle is an indication of the hydrophobicity of a surface; the greater the contact angle, the greater the hydrophobicity [33]. Changes in PE film hydrophobicity were determined by measuring the water contact angle (WCA) using a contact angle measuring device (JC2000P, Zhongchen, Shanghai, China) connected to a computer with image recording software. WCA was measured at five different regions of each plastic sample.

#### 2.4.2. Fourier Transform Infrared Spectroscopy Measurements

Changes in the carbonyl index of the PE films were analyzed using an FTIR spectrophotometer (NEXUS 670, Nicolet, Madison, WI, USA) with a CONTINUUM microscope. A film pressing method was used to analyze the biodegradation of PE samples. A polymer sample (approximately 1 cm × 1 cm) was pressed onto the bottom of the IR transmitting window, and IR spectra were obtained in absorbance mode. The PE films were analyzed at a 4 cm^−1^ resolution with 32 scans in the 4000–400 cm^−1^ range. For analysis, all spectra were processed off-line with OMNIC software in automatic baseline correction mode. The oxidation of the PE film was expressed as the carbonyl index, i.e., the ratio of the absorbance peak of carbonyl at 1712 cm^−1^ to that of the internal standard CH_2_ at 1462 cm^−1^ [34].

#### 2.4.3. Scanning Electron Microscopy Observations of Bacterial Biofilms

Biofilms on film sheets that had been exposed to individual bacterial strains and bacterial consortia were observed using scanning electron microscopy (SEM) (FEI Apreo S, Thermo Fisher Scientific, Waltham, MA, USA). PE test samples were dried in a desiccator for 24 h under a vacuum, cut into 5 mm × 5 mm pieces, sputter-coated with a gold layer (Q150R S, Quorum, Laughton, East Sussex, UK), and examined by SEM.

### 2.5. Carbon Dioxide Evolution

A modified apparatus based on the report by Li et al. [35] was designed to measure the CO_2_ evolution. In brief, each apparatus was made up of a flask containing the microbial culture, a filter to scrub CO_2_ from the incoming air supply, and a small beaker filled with 5 mL 0.05 M NaOH to absorb CO_2_ produced by microbial bioactivity. One hundred milliliters of microbial culture and 0.3 g of polymer were added to each test flask. The reaction solution was retrieved and replaced with the same volume of fresh NaOH solution every 15 days. The retrieved NaOH solutions were back-titrated with 0.01 M HCl, and the amount of absorbed CO_2_ was calculated. Gas-tight sealing of the vessels was performed to prevent water evaporation during the long incubation period and to minimize any escape of CO_2_. Medium with microbes but no polymer served as controls.

### 2.6. Weight Loss

To facilitate the accurate measurement of plastic film weight loss [36], the biofilm was first completely removed from the film surface with 2% *w*/*v* aqueous sodium dodecyl sulfate for 4 h using a rotary shaker, followed by rinsing with de-ionized water. The washed film was placed on filter paper and dried overnight at 40 °C for 12 h before weighing. The film weight loss was calculated as follows:

Percentage weight loss = ((Initial weight − Final weight)/Initial weight) × 100.

### 2.7. Strain Identification

The bacterial isolates that demonstrated potential PE degrading capability were identified by amplification of 16S rRNA with the universal primers 27F (5′-AGAGTTTGATCCTGGCTCAG-3′) and 1492R (5′-GGTTACCTTGTTACGACTT-3′). PCR amplifications were performed as follows: initial denaturing at 95 °C for 5 min; 25 cycles of denaturing at 95 °C for 30 s, annealing at 56 °C for 30 s, and extension at 72 °C for 1.5 min; and a final extension at 72 °C for 10 min. The reaction mixture (total 20 μL) contained 10× Ex Taq buffer (2.0 μL), 5 u Ex Taq (0.2 μL), 2.5 mM DNTP mix (1.6 μL), 1 μL of each primer (5 pmol), 13.7 μL water, and 0.5 μL of DNA sample. The resulting PCR products were sequenced by the Majorbio Bio-pharm Technology Co., Ltd. of Shanghai, China, and the isolated strain was identified using the Basic Local Alignment Search Tool (BLAST) of the National Center for Biotechnology Information (NCBI). The partial 16S rDNA nucleotide sequences of two PE degradation strains were submitted to GenBank at NCBI under the accession numbers SAMN16976735 and SAMN16976736.

### 2.8. Statistical Analysis

All experiments were repeated three times, and data were presented as the mean ± standard error (SE). Statistical analyses were performed using GenStat 18th edition (VSN International Ltd., Rothamsted, UK), and graphs were created in Origin 9.2 (OriginLab OriginPro 2015, Northampton, MA, USA). Comparisons among treatments were performed by analysis of variance (ANOVA) followed by Fisher’s protected least significant difference test.

## 3. Results

### 3.1. Screen for Polyethylene-Degrading Strains

The enrichment and preliminary screen for bacteria capable of degrading PE film was performed by incubating soil plastic residues in minimal liquid medium supplemented with UPF powder as the sole carbon source. To obtain as many bacteria as possible, two kinds of minimal medium with different nutrient compositions were used. At the end of a 90-day incubation period, three (C1–C3) and six (L1–L6) bacterial strains were ultimately obtained from Czapek–Dox and LCFB medium, respectively. Such long enrichment time indicated that these isolates might have the ability to use the PE as an energy source for cell growth. However, the possibility that carbon within the soil or plastic film residue might have contributed to bacterial growth could not be ruled out. Therefore, these isolates were further grown for another 90 days in the presence of UPF, and their effects on plastic film degradation properties such as weight loss, WCA, and carbonyl index were determined. The weight losses of plastic films were 0.22% (C1), 0% (C2), and 0% (C3) for the three isolates in Czapek-Dox medium and 0.25% (L1), 0.24% (L2), 0.25% (L3), 0% (L4), 0.23% (L5), and 0.49% (L6) for the six isolates in LCFBM medium. Although the film weight losses seemed relatively low, the presence of all isolates significantly decreased the WCA of the PE films (Figure S1). Changes in films carbonyl index varied depending on the isolates, with only C3 and L5–L6 showing a noticeable capability to increase this degradation property (Figure S2). Based on the degradation property changes, C3 and L6 were selected for further study. Subsequently, the C3 and L6 strains were identified as *Arthrobacter* sp. and *Streptomyces* sp., respectively, based on 16S rDNA sequencing.

### 3.2. Bacterial Growth on Polyethylene Film

**Figure 1:**
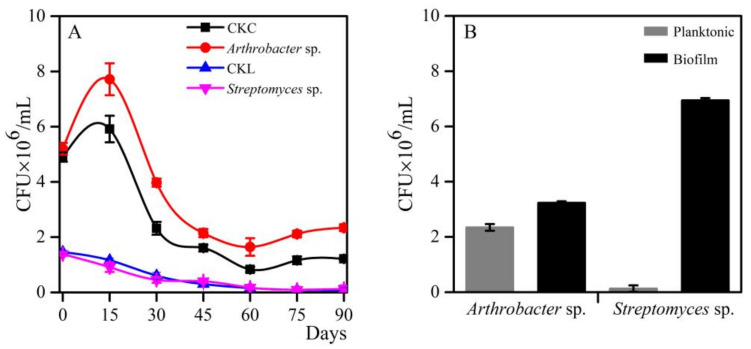
Growth and biofilm formation of *Arthrobacter* sp. and *Streptomyces* sp. in the presence of polyethylene (PE) films as a sole carbon source. (**A**) Colony-forming units (CFU) of planktonic bacteria. CKC and CKL indicate Czapek–Dox medium and liquid carbon-free basal medium without PE film and with *Arthrobacter* sp. and *Streptomyces* sp., respectively. (**B**) CFU of bacteria in planktonic culture and on biofilms formed on the surface of plastic film at day 90. Bars indicate the mean ± SE (n = 3).

Bacteria may degrade PE film into smaller carbon-containing compounds that can serve as substrates to support further bacterial growth. We therefore measured the numbers of bacteria adhering to the UPF surface and growing planktonically in the culture suspension by the serial dilution method in a long-term culture period up to 90 days. Compared with optical density measurements at 600 nm, the serial dilution method is more effective for measuring living microbes rather than plastic particles that may result from physical fragmentation. As shown in Figure 1A, the population density of the *Arthrobacter* sp. strain, as indicated by CFU/mL, was higher in the planktonic culture in the presence of PE plastic films, suggesting that PE could be used as an energy source, allowing greater establishment and reproduction by the inoculum. Furthermore, regardless of PE addition, the population density of *Arthrobacter* sp. peaked at day 15, decreased significantly until day 45, and then stabilized. Conversely, the growth of *Streptomyces* sp. in the planktonic phase was not substantially affected by the added PE, and its population density decreased to an undetectable level from the initial time point to day 60.

The number of living bacteria in suspension and on the surface of the PE film were determined at day 90 (Figure 1B). Both bacterial isolates showed higher CFUs on the film surface than in planktonic suspension, suggesting that they were able to form biofilms on PE film surfaces. The population density of *Arthrobacter* sp. on PE film surfaces was 1.4-fold higher than that in suspension. Surprisingly, strain *Streptomyces* sp. showed hardly any increase in growth in planktonic suspension, but a remarkably strong attachment to PE film surface, where its population density was approximately 50-fold higher. These results suggest that although both *Arthrobacter* sp. and *Streptomyces* sp. demonstrated a potential ability to degrade PE film (e.g., by forming a biofilm), the former strain lived planktonically in the medium, whereas the latter lived on the surface of the plastic film. Such distinct living environmental niches (planktonic vs. biofilm) also implied that these two strains likely belong to different metabolic types and could access different PE-derived nutritional compounds, raising the question of whether a combination of these strains as a bacterial consortium could more effectively degrade PE film.

### 3.3. Changes in Plastic Film Properties

**Figure 2:**
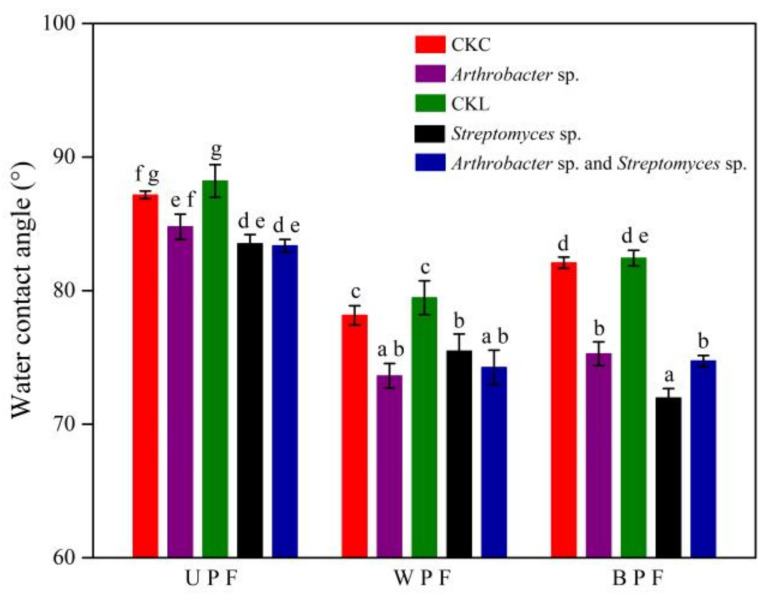
Effects of a 90-d bacterial incubation on the water contact angle (WCA) of polyethylene films. UPF (unpretreated plastic film). WPF (weathered plastic film). BPF (buried plastic film). CKC and CKL are control plastic films that were not incubated with bacteria in Czapek–Dox and liquid carbon-free basal medium, respectively. Bars indicate the mean ± SE (n = 3), and bars with different letters are significantly different (*p* < 0.05, two-way ANOVA and Fisher’s protected LSD test).

WCA can be used to assess changes in polymer surface hydrophobicity. Prior exposure of PE film to natural weathering and soil burial may aid the formation of microbial biofilms on its surface and promote its degradation. Therefore, besides UPF, WPF and BPF were also used for the measurement of WCA in the presence of individual bacterial isolates and a combined consortium (Figure 2). Without bacterial isolates, UPF had the highest WCA, followed by BPF and WPF. However, if these plastic films were incubated with microbes, their WCAs were decreased to varying degrees, indicating that the bacteria were capable of changing the hydrophobicity of PE film. In detail, the WCA of UPF, WPF, and BPF decreased by 2.7%, 5.8%, and 8.3% when exposed to *Arthrobacter* sp. and by 5.3%, 5.0%, and 12.7% for *Streptomyces* sp., respectively. The consortium of both strains decreased WCA of the three plastics by 5.5%, 6.6%, and 9.3%, respectively.

**Figure 3:**
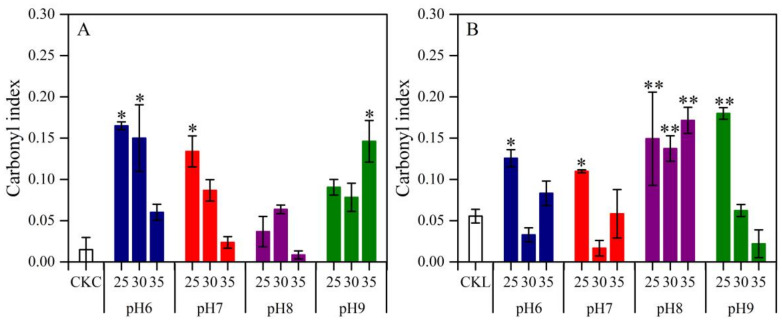
Effect of pH (6–9) and temperature (25–35 °C) on polyethylene film carbonyl index in the presence of *Arthrobacter* sp. (**A**) and *Streptomyces* sp. (**B**). CKC and CKL indicate polyethylene film without bacteria in Czapek–Dox and liquid carbon-free basal medium at 25 °C in pH 7, respectively. Bars indicate the mean ± SE (n = 3), and bars with asterisk differ significantly from CKC (**A**) or CKL (**B**). * *p* < 0.05, ** *p* < 0.01.

FTIR has been reported to be an effective method to evaluate the changes in PE chemical structure after biodegradation by selected microorganisms [37]. As shown in Figure 1, *Arthrobacter* sp. and *Streptomyces* sp. were grown in the different physical phases, suggesting that these two bacterial strains probably belong to distinctive metabolic types with different excretion of enzymes to attack the chemical bonds of PE film. Therefore, whether they demonstrate differential physiological conditions to transform these chemical bonds (as indicated by carbonyl index) was tested. As shown in Figure 3, the greater carbonyl index was observed under temperatures ranging from 25 to 30 °C for both strains. However, *Arthrobacter* sp. was more effective in the modification of chemical bonds of PE under neutral or slightly acidic conditions (pH6), whereas *Streptomyces* sp. was more effective at weak alkaline conditions (pH8). These results suggest that environmental conditions such as pH can influence the degrading process of PE by these bacteria, and further prove that both strains have different metabolic functions.

**Figure 4:**
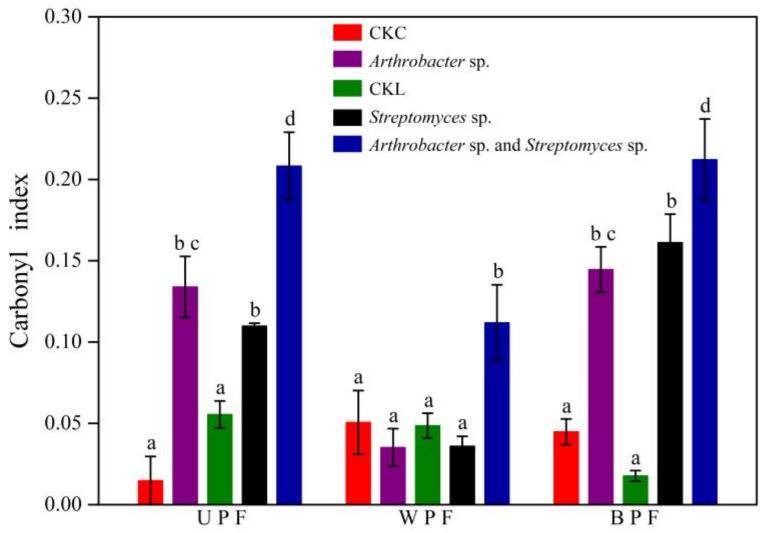
Effects of bacterial incubation for 90 days on the carbonyl indices of polyethylene films. UPF, WPF, and BPF are defined in Figure 2. The abbreviations CKC and CKL are also the same as those used in Figure 2. Bars indicate the mean ± SE (n = 3), and bars with different letters are significantly different (*p* < 0.05, two-way ANOVA and Fisher’s protected LSD test).

Under these physiological conditions, the effects of bacterial inoculum on the carbonyl index of three kinds of PE films (UPF, WPF, and BPF) were determined. As shown in Figure 4, the carbonyl indices of UPF exposed to individual strains of *Arthrobacter* sp. and *Streptomyces* sp. were, respectively, 9.0 times and 2.0 times higher than those of their corresponding uninoculated controls. For BPF, these values were 3.2 times and 9.1 times higher, respectively. Interestingly, inoculation with the individual strains had no effect on the carbonyl index of WPF. However, the carbonyl indices of all films increased dramatically after inoculation with the bacterial consortium of both strains. The carbonyl indices of UPF, WPF, and BPF were 3.8, 2.3, and 23.4 times higher, respectively, than their corresponding controls. Hence, it was clear that the microbial consortium exhibited better degradation ability than either of its constituent microorganism. It is most likely that such remarkable alternation of PE chemical structure resulted from the complementary metabolic effect of both strains.

### 3.4. Scanning Electron Microscopy Observation

**Figure 5:**
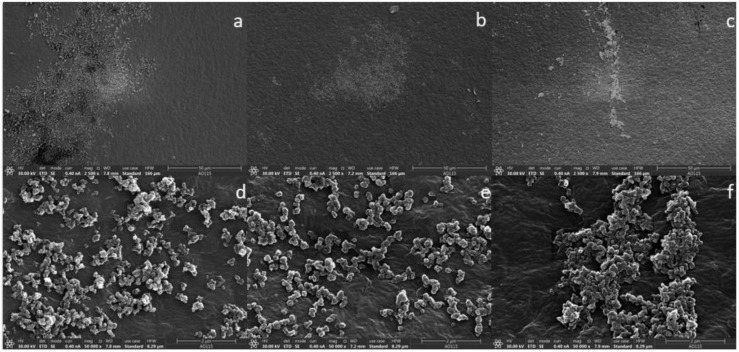
SEM micrographs of *Arthrobacter* sp. attached to the polyethylene film surface. Unpretreated plastic film (UPF) at 2500× magnification (**a**) and 20,000× magnification (**d**). Weathered plastic film (WPF) at 2500× magnification (**b**) and 20,000× magnification (**e**). Buried plastic film (BPF) at 2500× magnification (**c**) and 20,000× magnification (**f**).

**Figure 6:**
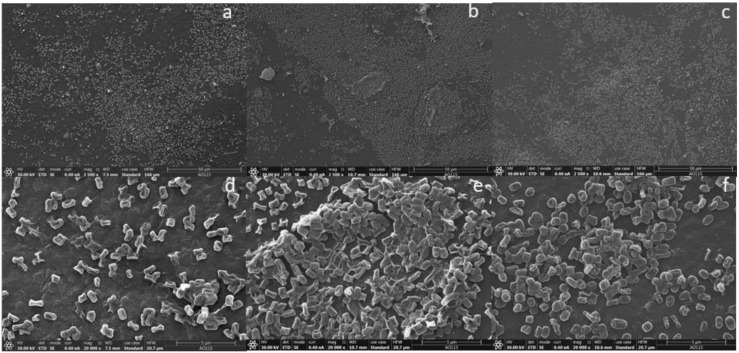
SEM micrographs of *Streptomyces* sp. attached to the polyethylene film surface. Unpretreated plastic film (UPF) at 2500× magnification (**a**) and 20,000× magnification (**d**). Weathered plastic film (WPF) at 2500× magnification (**b**) and 20,000× magnification (**e**). Buried plastic film (BPF) at 2500× magnification (**c**) and 20,000× magnification (**f**).

**Figure 7:**
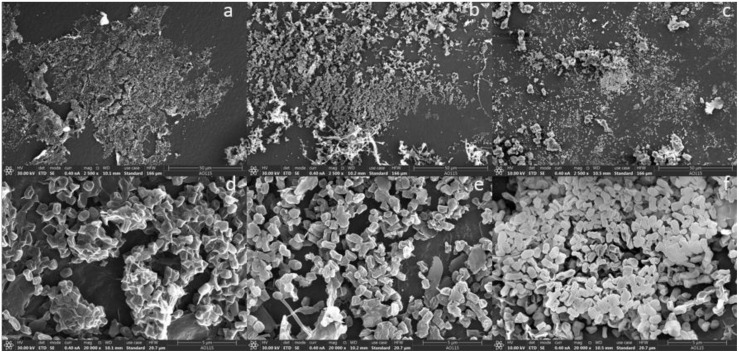
SEM micrographs of a bacterial consortium (*Arthrobacter* sp. and *Streptomyces* sp.) attached to the polyethylene film surface. Unpretreated plastic film (UPF) at 2500× magnification (**a**) and 20,000× magnification (**d**). Weathered plastic film (WPF) at 2500× magnification (**b**) and 20,000× magnification (**e**). Buried plastic film (BPF) at 2500× magnification (**c**) and 20,000× magnification (**f**).

The attachment of microbes to the three PE film (UPF, WPF, and BPF) surfaces were investigated by SEM. *Arthrobacter* sp. colonized the PE surface and formed a three-dimensional structure, although some portions of the surface were relatively less colonized, indicating that the ability of this strain to develop a biofilm was quite weak (Figure 5). On the other hand, *Streptomyces* sp. uniformly aggregated on the plastic surface and formed multicellular structures, producing a three-dimensional biofilm that was surrounded by a single-layer biofilm; however, this bacterium was also more widely dispersed over the surface (Figure 6). Overall, both bacterial strains were capable of developing a biofilm on the film surface with different colonizing ability, consistent with bacterial growth quantified by CFUs in Figure 1B. Surprisingly, the consortium formed much denser, multi-layered biofilms, with bacteria more widely distributing across the film surface (Figure 7). Taken together, these results show that as compared with single strains, the bacterial consortium formed a much greater bacterial attachment on the PE surface, where the formation of biofilm is a critical initial step in the biodegradation process of PE.

### 3.5. Carbon Dioxide Evolution

**Figure 8:**
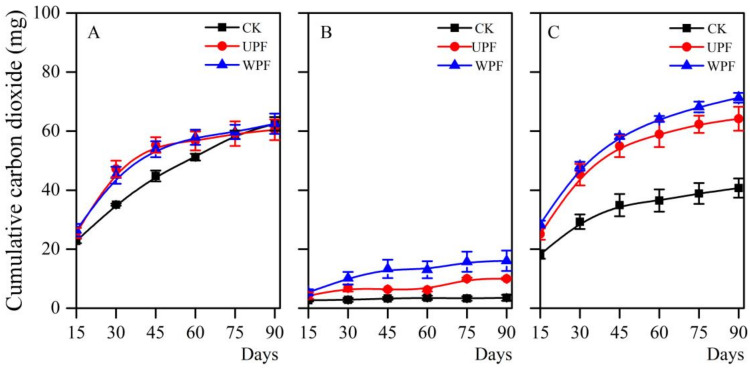
Cumulative CO_2_ evolution from polyethylene film incubated with individual bacterial strains and a bacterial consortium, measured at 15-day intervals over 90 days. (**A**) *Arthrobacter* sp., (**B**) *Streptomyces* sp., (**C**) *Arthrobacter* sp. and *Streptomyces* sp. CK indicates the presence of bacteria without polyethylene film. Data are the mean ± SE (n = 3).

CO_2_ is the final metabolic product of PE that was provided as the sole carbon source, and its evolution was therefore monitored (Figure 8). Compared with the presence of bacteria only, the addition of PE (UPF or WPF) led to much higher CO_2_ evolution for strain *Arthrobacter* sp., particularly during the experiment period of 60 days (Figure 8A) and a maximum of about 60 mg of CO_2_ cumulated was detected. More CO_2_ was also observed to accumulate when the *Streptomyces* sp. strain was grown with PE film (Figure 8B). Cumulative CO_2_ evolution of 10.0 ± 0.4 and 16.1 ± 3.5 mg was detected for UPF and WPF, respectively, and was very low only in the presence of single bacterial species grown without film (3.5 ± 1.0 mg). Surprisingly, the rate and magnitude of CO_2_ evolution were very high for the bacterial consortium (Figure 8C). Cumulative CO_2_ of 64.2 ± 4.0 and 71.3 ± 1.7 mg were detected for UPF and WPF, respectively, and 40.7 ± 3.3 mg for the only consortium bacteria grown without film. These results provide strong evidence that the microbial consortium is more effective at PE degradation than either of its constituent microorganisms. Considering that these great differences might be caused by functional masterbatch of PE that is an easily utilized carbon-containing component, the CO_2_ evolution from individual LLDPE, LDPE, or functional masterbatch were determined. Result showed that CO_2_ evolution was also markedly higher for the LLDPE or LDPE (Figure S3), suggesting that PE, not the functional masterbatch, was responsible for the enhanced CO_2_ evolution driven by the bacteria.

## 4. Discussion

Although the durability of plastic is one of the important qualities to be considered for practical usage, nonetheless, it presents a major problem in terms of environmental pollution. Microbial degradation is one of the best options for the eco-friendly disposal of these plastic wastes. In this study, two novel bacterial strains with strong PE degradation capabilities were isolated. Their 16S rRNA gene sequences showed 99.72% and 99.37% identity to those of *Arthrobacter* sp. and *Streptomyces* sp. Some bacterial species within these genera have been reported to degrade PE [38,39], confirming that many bacteria belonging to these two genera have the actual potential to degrade PE. However, further characterization is needed for complete taxonomic classification and to determine how closely they are related to the bacteria in the present study.

Because most bacterial surfaces are hydrophilic, the hydrophobicity of PE interferes with bacterial adhesion to PE surfaces [40]. It is well known that microbial degradation of PE requires bacterial adhesion and subsequent biofilm formation on the surface, where the bacteria survive in low-nutrient environments and utilize solid substrates [20]. In this study, *Arthrobacter* sp. and *Streptomyces* sp. CFUs were approximately 1.4 times and 53.7 times higher, respectively, for biofilm-formed cells than for planktonic cells (Figure 1B), indicating that both isolates had a propensity for attachment to PE film, particularly the latter. Moreover, *Arthrobacter* sp. mainly grows in a planktonic manner, while *Streptomyces* sp. propagated on the surface. Such phenomenon of imbalanced growth between bacteria in biofilms and the planktonic state was previously reported by Sivan [20]. In contrast to planktonic microbes, attached microbes can secrete degradation enzymes and auxiliary molecules near the PE surface, maximizing their concentration around the polymer and increasing the probability of substrate modification [24]. Biofilm formation has been considered to be firmly linked with bacteria-produced exopolysaccharides [41]. The superiority growth of *Arthrobacter* sp. in the planktonic phase (Figure 1A) suggests that this strain might access some soluble unknown PE-derived compounds more easily, but its capability to synthesize exopolysaccharides was probably very weak.

WCA is widely used as an indicator of plastic hydrophobicity. A lower contact angle indicates a greater hydrophilicity of surfaces that are more easily colonized by microorganisms. Some studies have shown that microbial attachment and biofilm formation can alter the change from a hydrophobic to a hydrophilic characteristic of plastics film surfaces [21]. In the presence of bacteria, all the PE surfaces became more hydrophilic, as shown by the significantly decreased WCA (Figure 2), indicating that the attachment of our isolated bacterial strain could in turn intensify the hydrophilic process, which would facilitate microbes to transform the chemical bonds in the following stage of the biodegradation process.

Monitoring the formation and disappearance of carbonyl peaks with FTIR is essential for understanding the mechanisms of plastic biodegradation. Special emphasis is placed on the carbonyl index, i.e., the ratio of the absorbance peak at 1712 cm^−1^ to that of CH_2_ at 1462 cm^−1^. The carbonyl indexes of PE films were determined under varying temperatures and pH values in order to optimize the physiologic conditions for biodegradation. Conditions were favorable and relatively stable over a range of temperatures (25–30 °C) (Figure 3), but were differentially affected by pH, with relative high carbonyl index at a neutral or slightly acidic pH point for *Arthrobacter* sp. and a slightly alkaline one for *Streptomyces* sp. Previous studies conducted by Goel et al. [42] and Balasubramanian et al. [38] showed that bacterial strains of *Arthrobacter* sp. not only grew best at 30 ± 0.2 °C and pH 5.0 ± 0.2, but also exhibited the ability to effectively degrade polycarbonate and PE, whereas *Streptomyces* sp. effectively degraded PE at 30 °C under pH 7.5 [43]. Our results were in agreement with these reports, but more importantly, the differences in growth of the two strains in different physiologic conditions (e.g., pH) and growing microenvironments (planktonic vs. surface) indicated that they belong to different metabolic groups, and that a mixture of the strains led to more effective degradation of PE. Furthermore, manipulation of physiologic conditions may be an important factor for promoting efficient bio-degradation of plastic film.

In this study, we showed that the carbonyl index of UPF, an intact plastic film, increased following exposure to bacterial isolates (Figure 4). This change in film surface chemistry may be beneficial for further attachment of bacteria, thereby enhancing the biodegradation of PE mulch films. Yang et al. [16] found that PE inoculated with bacteria *Bacillus* sp. YP1 and *Enterobacter asburiae* YT1 showed new carbonyl peaks. Balasubramanian et al. [38] reported that the keto carbonyl, ester carbonyl, vinyl bond, and internal double bond indices of high-density PE increased after treatment with *Arthrobacter* sp. The results of our study are consistent with the studies cited above, confirming that an increase in carbonyl index can be used as a marker for biodegradation of PE.

CO_2_ evolution has been used previously to evaluate the biodegradability of PE. In this study, the addition of PE led to much higher amounts of CO_2_ produced (Figure 8), providing evidence that the substrates could be mineralized by the bacterial isolates as a carbon and energy source. It is consistent with some previous reports showing that pure bacterial isolates could metabolize the PE as the final CO_2_. Abraham et al. [44] showed that CO_2_ evolution from the biodegradation of PE pieces reached 2.85 g/L in the presence of *Aspergillus nomius* and 4.27 g/L for *Streptomyces* sp. Likewise, Shah et al. [45] measured CO_2_ evolution of 1.85 g/L by *Fusarium* sp. over a 30-day period with LDPE film pieces, and 0.45 g/L for control with no film. In this study, the addition of PE led to higher CO_2_ evolution than the control, likely because carbonyl groups (Figure 4) were assimilated and decomposed by microbes, leading to higher plastic film mineralization and maintaining the higher bacterial viability (Figure 1) and then a higher metabolic turnover of these viable bacteria to produce more CO_2_.

In this study, the biodegradation properties of naturally occurring WPF and BPF were compared with those of UPF. We showed that both *Arthrobacter* sp. and *Streptomyces* sp. were unable to increase the carbonyl index of WPF (Figure 4). This was inconsistent with the study by Syranidou et al. [46] who observed an increase in the functional groups of WPF surfaces when exposed to microorganisms. The discrepancy might be explained by the fact that WPF (10 μm) is thicker than UPF and BPF (6 μm). Thick film has been reported to be degraded at a slower rate due to higher relative surface area [47,48]. However, compared with UPF, the surface hydrophilicity of WPF and BPF increased (Figure 2), and higher cumulated CO_2_ production was observed (Figure 8). Likewise, Novotný et al. [25] reported that virgin LLDPE was not attacked by a *Bacillus amyloliquefaciens* strain, whereas pretreated LLDPE deteriorated after bacterial treatment. Hence, pretreatments like weathering and soil burial prior to microbial incubation may increase the susceptibility of polymer biodegradation.

Until now, most studies have focused on the use of single organisms to degrade PE as a convenient way to investigate metabolic pathways, but these studies ignore the possibility that efficient PE biodegradation might result from the cooperative processes of mixed species. Our results suggest that plastic degradation is enhanced by the mutualistic activity and co-metabolism of two bacterial strains. Besides effectively coordinating activities of the microbial mixture to alter the PE degradation properties and to metabolize the PE as CO_2_, bio-film formation on the surface was also observed by SEM micrographs. The images showed that more extensive biofilms were formed with the bacterial consortium than either individual isolate (Figure 5, Figure 6 and Figure 7). This may explain the higher carbonyl index (Figure 4) and the greater CO_2_ production (Figure 8 and Figure S3) exhibited by the consortium. These results suggest that microbial communities could effectively degrade PE in a coordinated manner. Why and how these two strains cooperatively or complementarily act in PE remains unknown. Genetic information based on next generation sequencing may address this interesting and important issue in the future. However, the present study still has deepened our understanding of PE biodegradation by the two strains, which will be valuable bioresources.

## 5. Conclusions

Microbial biodegradation holds promise for the remediation of soil plastic pollution caused by the widespread use of agricultural mulch films. We isolated and identified two bacterial strains capable of growing on PE film as a sole carbon source. Individually, both strains formed biofilms on PE film, decreased film hydrophobicity, increased film carbonyl index, and mineralized PE film. Together, they formed thicker and more complex biofilms, had much greater effects on film hydrophobicity and surface chemistry, and promoted higher rates of PE mineralization. Our results demonstrate that plastic degradation can be enhanced by the combined activities of bacteria in a mixed population.

## Supplementary Materials

The following are available online at https://www.mdpi.com/2076-2607/8/12/1979/s1, Figure S1: Isolated bacterial strains decreased the water contact angle of polyethylene film in liquid medium. CCK and LCK are control plastic film that were not incubated with bacteria in Czapek–Dox and liquid carbon-free basal medium, respectively. C1–C3 and L1–L6 are unpretreated PE films incubated with individual strains. Bars indicate the mean ± SE (n = 3), and bars with different letters are significantly different (*p* < 0.05, one-way ANOVA and Fisher’s protected LSD test), Figure S2: Changes in the carbonyl index of polyethylene films incubated with individual bacterial strains in liquid medium. The designations CCK and LCK are the same as in Figure S1. C1–C3 and L1–L6 are described in Figure S1. Bars indicate the mean ± SE (n = 3), and bars with different letters are significantly different (*p* < 0.05, one-way ANOVA and Fisher’s protected LSD test), Figure S3: Cumulative CO_2_ evolution from LLDPE, LDPE, and functional masterbatch powder incubated with individual bacterial and a bacterial consortium, measured at 15-day intervals over 90 days. A, *Arthrobacter* sp., B, *Streptomyces* sp., C, *Arthrobacter* sp. and *Streptomyces* sp. CK indicates the presence of bacteria without polyethylene film powder. LLDPE, linear low-density polyethylene. LDPE, low-density polyethylene. Data are the mean ± SE (n = 3).
