# Supplementary material for: Greater Biofilm Formation and Increased Biodegradation of Polyethylene Film by a Microbial Consortium of Arthrobacter sp. and Streptomyces sp
Source: Microorganisms. 2020 Dec 12;8(12):1979. doi: 10.3390/microorganisms8121979 (PMC7764375; doi:10.3390/microorganisms8121979)
Supplement: Supplementary file 1 [file microorganisms-08-01979-s001.pdf]

Supplementary material

Greater Biofilm Formation and Increased Biodegradation of Polyethylene Film by a Microbial Consortium of *Arthrobacter* sp. and *Streptomyces* sp.

Ya-Nan Han [1,2,3], Min Wei [1], Fang Han [1], Chao Fang [4], Dong Wang [1], Yu-Jie Zhong [1], Chao-Li Guo [1], Xiao-Yan Shi [1], Zhong-Kui Xie [2,3] and Feng-Min Li [1,*]

1 State Key Laboratory of Grassland Agro-ecosystems, Institute of Arid Agroecology, School of Life Sciences, Lanzhou University, Lanzhou 730000, China; hanyn15@lzu.edu.cn (Y.-N.H.); weim@lzu.edu.cn (M.W.); hanf19@lzu.edu.cn (F.H.); wangd16@lzu.edu.cn (D.W.); zhongyj19@lzu.edu.cn (Y.-J.Z.); guochl19@lzu.edu.cn (C.-L.G.); shixiaoyan@lzu.edu.cn (X.Y.S.)

2 Northwest Institute of Eco-environment and Resources, Chinese Academy of Sciences, No. 320 West Donggang Road, Lanzhou, Gansu 730000, China; wxhcas@lzb.ac.cn (Z.-K.X.)

3 University of Chinese Academy of Sciences, Beijing 100049, China

4 Institute of Ecology, School of Applied Meteorology, Nanjing University of Information Science and Technology, Nanjing 210044, China; fangch12@lzu.edu.cn (C.F.)

* Correspondence: fmli@lzu.edu.cn (F.-M.L.)

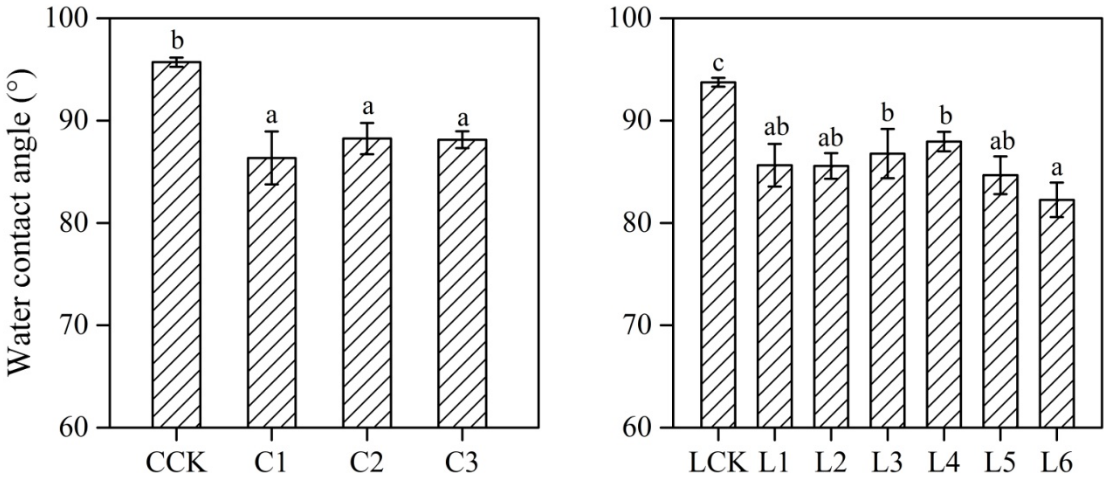

Figure S1. Isolated bacterial strains decreased the water contact angle of polyethylene film in liquid medium. CCK and LCK are control plastic film that were not incubated with bacteria in Czapek–Dox and liquid carbon-free basal medium, respectively. C1–C3 and L1–L6 are unpretreated PE films incubated with individual strains. Bars indicate the mean ± SE (n = 3), and bars with different letters are significantly different ($p < 0.05$, one-way ANOVA and Fisher's protected LSD test).

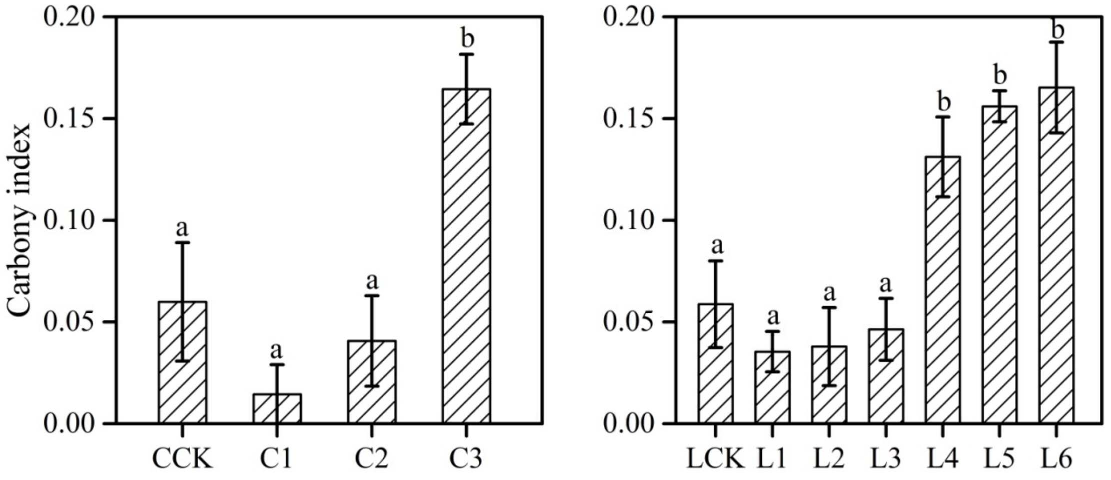

Figure S2. Changes in the carbonyl index of polyethylene films incubated with individual bacterial strains in liquid medium. The designations CCK and LCK are the same as in Figure S1. C1–C3 and L1–L6 are described in Figure S1. Bars indicate the mean ± SE (n = 3), and bars with different letters are significantly different (p < 0.05, one-way ANOVA and Fisher's protected LSD test).

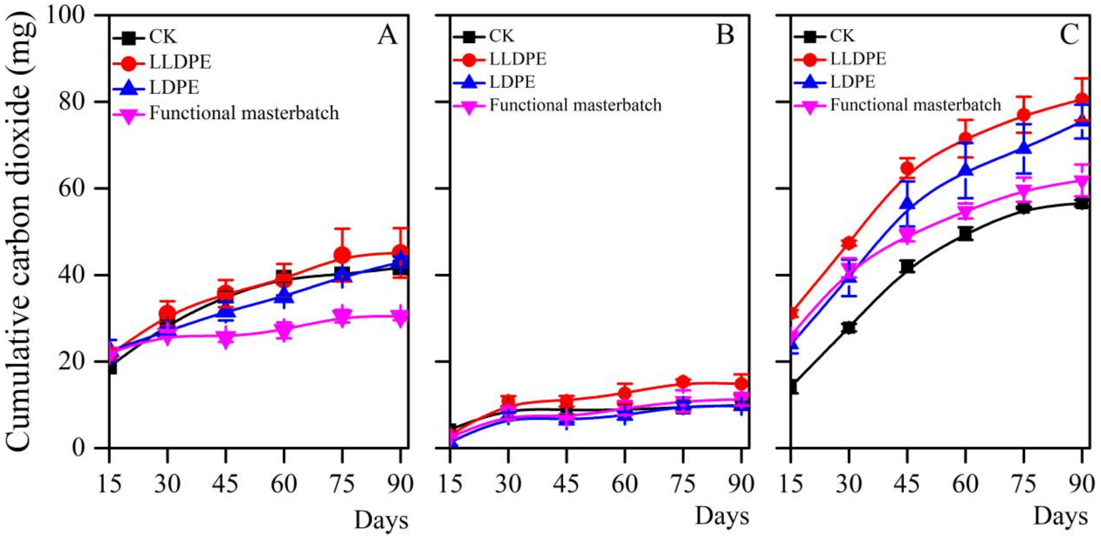

Figure S3. Cumulative CO_2 evolution from LLDPE, LDPE and functional masterbatch powder incubated with individual bacterial and a bacterial consortium, measured at 15-day intervals over 90 days. A, *Arthrobacter* sp., B, *Streptomyces* sp., C, *Arthrobacter* sp. and *Streptomyces* sp. CK indicates the presence of bacteria without polyethylene film powder. LLDPE, linear low-density polyethylene. LDPE, low-density polyethylene. Data are the mean ± SE (n = 3).
